# Muscle-Bone Crosstalk and Metabolic Dysregulation in Children and Young People Affected with Type 1 Diabetes: Mechanisms and Clinical Implications

**DOI:** 10.3390/cells14201611

**Published:** 2025-10-16

**Authors:** Rossella Vitale, Giovanna Linguiti, Vanja Granberg, Crescenza Lattanzio, Paola Giordano, Maria Felicia Faienza

**Affiliations:** 1 Pediatric Unit, Department of Precision and Regenerative Medicine and Ionian Area, University of Bari “Aldo Moro”, Piazza G. Cesare 11, 70124 Bari, Italy; 2 Pediatric Unit “B. Trambusti”, Department of Interdisciplinary Medicine, University of Bari “Aldo Moro”, Piazza G. Cesare 11, 70124 Bari, Italy

**Keywords:** diabetes type 1, bone mineral density, myopathy, glycemic variability

## Abstract

Pediatric type 1 diabetes (T1D) disrupts musculoskeletal development during critical windows of growth, puberty, and peak bone mass accrual. Beyond classic micro- and macrovascular complications, accumulating evidence shows a dual burden of diabetic bone disease—reduced bone mineral density, microarchitectural deterioration, and higher fracture risk—and diabetic myopathy, characterized by loss of muscle mass, diminished strength, and metabolic dysfunction. Mechanistically, chronic hyperglycemia, absolute or functional insulin deficiency, and glycemic variability converge to suppress PI3K–AKT–mTOR signaling, activate FoxO-driven atrogenes (atrogin-1, MuRF1), and impair satellite-cell biology; advanced glycation end-products (AGEs) and RAGE signaling stiffen extracellular matrix and promote low-grade inflammation (IL-6, TNF-α/IKK/NF-κB), while oxidative stress and mitochondrial dysfunction further compromise the bone–muscle unit. In vitro, ex vivo, and human studies consistently link these pathways to lower BMD and trabecular/cortical quality, reduced muscle performance, and increased fractures—associations magnified by poor metabolic control and longer disease duration. Prevention prioritizes tight, stable glycemia, daily physical activity with weight-bearing and progressive resistance training, and optimized nutrition (adequate protein, calcium, vitamin D). Treatment is individualized: supervised exercise-based rehabilitation (including neuromuscular and flexibility training) is the cornerstone of skeletal muscle health. This review provides a comprehensive analysis of the mechanisms underlying the impact of type 1 diabetes on musculoskeletal system. It critically appraises evidence from in vitro studies, animal models, and clinical research in children, it also explores the effects of prevention and treatment.

## 1. Introduction

Type 1 diabetes mellitus (T1D) is a chronic autoimmune disorder characterized by immune-mediated destruction of pancreatic β-cells, leading ultimately to an absolute insulin deficiency, and lifelong requirement of exogenous insulin. Although the disease can occur at any age, its peak onset occurs in childhood and adolescence, with a steadily increasing incidence. According to the International Diabetes Federation, an estimated 130,000 cases of type 1 diabetes are diagnosed worldwide each year in people under the age of 20 [1]. Advances in diabetes management, including subcutaneous insulin infusion systems, and continuous glucose monitoring technologies, have significantly improved both survival and quality of life in T1D individuals [2,3]. Nonetheless, self-management of the disease remains highly demanding, and many patients fail to achieve and sustain the level of glycemic control required to prevent acute complications, such as diabetic ketoacidosis and severe hypoglycemia, as well as long-term consequences [4]. Classically, the long-term complications of T1D have been categorized into microvascular (retinopathy, nephropathy, neuropathy) and macrovascular (atherosclerosis, cardiovascular disease) outcomes [5]. However, over the past two decades, a growing body of evidence has highlighted that T1D also negatively impacts the musculoskeletal system [6]. This negative impact includes both diabetic bone disease (DBD)—characterized by reduced bone mineral density, altered bone microarchitecture and increased risk of fractures and diabetic myopathy, which involves loss of muscle mass, reduced strength and impaired metabolic and regenerative capacity [6,7].

Childhood, and particularly adolescence, represent crucial periods for skeletal maturation, during which up to 40% of total bone mineral content is accrued. The pediatric population with T1D are particularly vulnerable to musculoskeletal deterioration because skeletal muscle and bone develop in close coordination during growth. The “muscle–bone unit” concept emphasizes the mechanical and biochemical crosstalk between these tissues, with muscle contraction providing mechanical loading for bone strength, and bone-derived factors influencing muscle metabolism [8].

This narrative review aims to highlight the current evidence on the cellular mechanisms underlying the muscle skeletal and bone impairment in T1D, focusing on in vitro, ex vivo, and human studies. Finally, we provide a perspective on potential strategies for prevention and treatment.

## 2. Cellular Mechanism of Muscle Skeletal Impairment

Chronic hyperglycemia is a key causal factor in the development of muscle impairment through a complex network of cellular and molecular mechanisms.

### 2.1. Impairment of Satellite Cells

**Figure 1:**
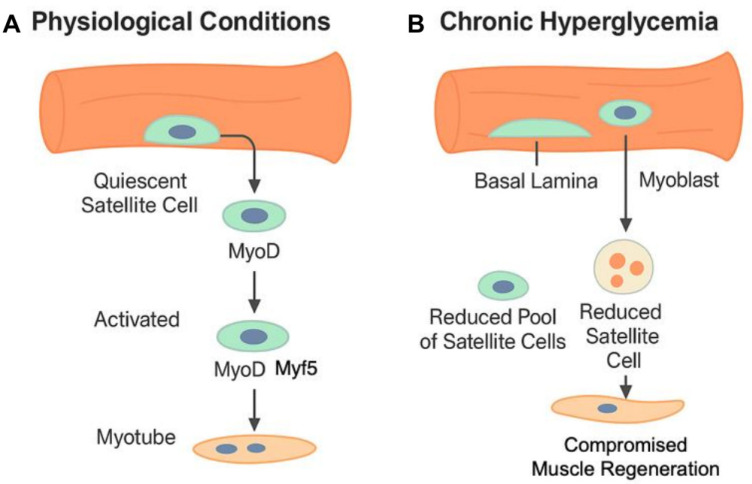
Regulation of satellite cells under physiological and chronic hyperglycemia conditions. Schematic representation of satellite cell behavior under physiological conditions (**A**) and chronic hyperglycemia (**B**). (**A**) In normal conditions, satellite cells reside quiescently between the sarcolemma and basal lamina and express Pax7. Upon muscle injury, they become activated, express MyoD and Myf5, proliferate as myoblasts, and undergo either differentiation into myotubes or asymmetric division to maintain the stem cell pool. (**B**) Under chronic hyperglycemia, reduced MyoD expression impairs proliferation and differentiation, leading to a diminished pool of satellite cells. This results in compromised muscle regeneration and progressive loss of muscle mass.

Chronic hyperglycemia impairs the biology and function of muscle stem cells (satellite cells). These cells are located between the muscle fiber membrane (sarcolemma) and the basal lamina. Under physiological conditions, satellite cells remain in a quiescent state; however, upon muscle injury, they become activated and initiate the myogenic process. Both quiescent and activated satellite cells express the transcription factor Pax7 (Paired Box 7), which is essential for preserving their identity and ensuring proper regenerative potential. Once activated, satellite cells begin to express MyoD and Myf5, transcription factors essential for myogenesis, and proliferate as myoblasts. A portion of these cells undergo asymmetric division to maintain the stem cell pool (self-renewal) [9,10]. The transcription factor MyoD triggers the transcription of myogenin and other muscle-specific genes, thus acting as a key regulator of myogenesis [11]. In condition of chronic hyperglycemia, a decrease in MyoD expression is observed; this downregulation is indicative of reduced myogenic potential, leading to impaired muscle regeneration due to a diminished number of satellite cells capable of self-renewal and proliferation. Over time, this compromises the maintenance and regeneration of muscle mass [9,12] (Figure 1).

### 2.2. Insulin Alteration

**Figure 2:**
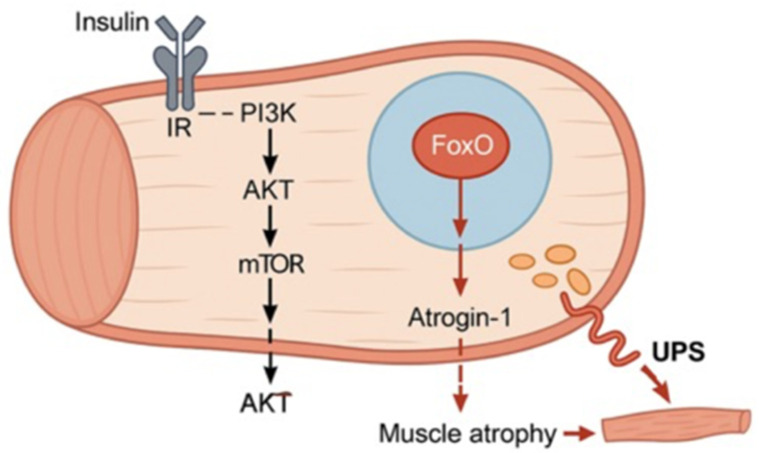
Insulin signaling impairment drives muscle atrophy in T1D.

Insulin is essential to stimulate glucose uptake and protein synthesis in skeletal muscle [13]. Insulin deficiency results in a higher rate of muscle protein degradation compared to synthesis. Patients with T1D exhibit impaired satellite cell repair capacity, and compromised muscle function. Normally, the insulin receptor (IR) and the insulin-like growth factor 1 receptor (IGF1R) regulate various cellular functions via the PI3K/AKT signaling pathway [14]. Insulin-like growth factor 1 (IGF-1) activates the PI3K/AKT pathway, which subsequently stimulates mTOR activity which positively regulates ribosomal protein S6 kinase (p70S6K) [15]. Insulin and IGF-1 stimulate proliferation and protein synthesis in myoblasts, whereas in mature myotubes the PI3K/mTOR/p70S6K axis remains crucial but is activated by different stimuli. Physiologically, this process increases protein synthesis and promotes muscle growth. During glucose uptake and protein synthesis, protein kinase B (AKT) activation in response to insulin or IGF-1 phosphorylates Forkhead box O (FoxO) transcription factors (FoxO1, FoxO3, and FoxO4), thereby inhibiting their transcriptional activity [16]. In insulin-deficient states, FoxO factors translocate into the nucleus and activate transcription of genes such as Mafbx (Muscle Atrophy F-box, also known as Atrogin-1) and MuRF1 (Muscle-specific RING Finger protein 1) [16]. Dephosphorylation of FoxO and increased expression of Mafbx and MuRF1 accelerate protein degradation, promoting muscle atrophy [17]. Although AKT can inhibit the ubiquitin-proteasome system (UPS) [17,18], it remains inactive in the absence of insulin or IGF-1. FoxO family members translocate to the nucleus, inducing UPS gene transcription and initiating protein degradation [19,20]. The UPS is the principal intracellular protein degradation mechanism, comprising an enzymatic cascade that tags proteins with ubiquitin for proteasomal degradation [20]. E3 ubiquitin ligases of the cullin-RING family, including MuRF1 and Mafbx/Atrogin-1, represent the largest known class of E3 ligases [21]. These ligases are significantly overexpressed in skeletal muscle during atrophy, actively contributing to the degradation of contractile and structural proteins [22] (Figure 2).

Schematic illustration of insulin signaling impairment under chronic hyperglycemia (T1D). Insulin deficiency leads to inactivation of the PI3K/AKT/mTOR pathway, preventing AKT-mediated inhibition of FoxO. Active FoxO translocates to the nucleus and induces the expression of Atrogin-1 and MuRF1, which activate the ubiquitin–proteasome system (UPS), resulting in enhanced protein degradation and muscle atrophy.

### 2.3. Accumulation of Advanced Glycation End-Products (AGEs)

Hyperglycemia leads to increased production of Advanced Glycation End-products (AGEs) due to the non-enzymatic glycation of macromolecules such as proteins, lipids, and nucleic acids by reducing sugars (Maillard reaction) [21,22]. AGEs activate multiple intracellular signaling pathways through binding to the transmembrane receptor for advanced glycation end-products (RAGE); these include PKC (Protein Kinase C), PI3K/Akt (Phosphoinositide 3-Kinase/Protein Kinase B), MAPK/ERK (Mitogen-Activated Protein Kinase/Extracellular signal-Regulated Kinase), and JAK/STAT (Janus Kinase/Signal Transducer and Activator of Transcription). This complex signaling network leads to increased nuclear factor kappa B (NF-κB) activity and reactive oxygen species (ROS) production [23]. RAGE is expressed in various cell types including inflammatory cells, endothelial cells, smooth muscle cells, neurons, osteoblasts, and osteoclasts. The interaction of AGEs with RAGE directly influences cellular function and metabolism via inflammatory processes and oxidative damage [24]. Intracellular oxidative stress can further amplify AGE production, perpetuating a deleterious feedback loop in target cells.

RAGE expression is found in satellite cells, and in myoblasts [25]. However, RAGE is typically absent in mature muscle fibers, but it is upregulated following acute injury or in cancer cachexia. Furthermore, RAGE-mediated signaling plays a role in physiological muscle turnover.

In pathological conditions characterized by excessive AGE accumulation, such as diabetes, AGE-RAGE signaling predominates over other pathways [26]. AGE-RAGE interaction stimulates phosphorylation of NF-κB, enhancing the expression of extracellular matrix (ECM) genes (e.g., type I collagen) and the production of pro-inflammatory cytokines such as tumor necrosis factor-α (TNF-α), interleukin-1β (IL-1β), and interleukin-6 (IL-6) [27]. Beyond skeletal muscle, AGEs also accumulate within the bone matrix, where cros-linking of type I collagen impairs biomechanical properties, leading to reduced elasticity and increased fragility. Excessive collagen accumulation results in fibrosis and ECM thickening, which both disrupt skeletal muscle structure and function and impair the proliferation and differentiation capacity of satellite cells., as demonstrated by Serban et al. [27]. Simultaneously, AGE accumulation is associated with a stiffer ECM that is more resistant to proteolytic enzymes in skeletal muscle, negatively impacting muscle strength and regenerative capacity, increasing tissue rigidity and damaging the ECM [28,29,30].

**Figure 3:**
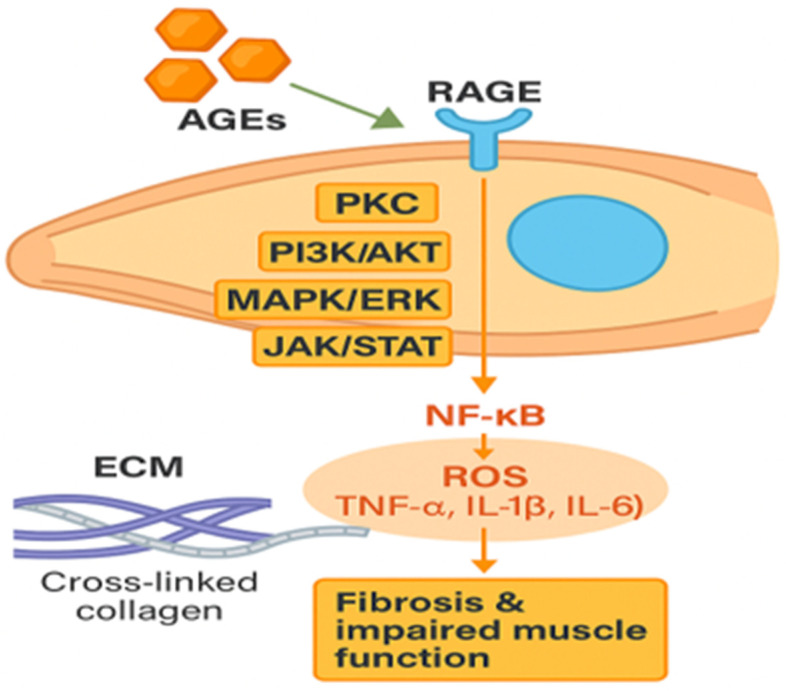
Molecular consequences of AGEs accumulation in skeletal muscle.

Moreover a study by Suzuki et al. [31] demonstrated that AGEs induce ROS formation within smooth muscle cells (myoblasts and myotubes via activation of the protein kinase C alpha (PKCα)/p47phox (a cytosolic subunit of the NADPH oxidase complex)/NADPH oxidase 2 (NOX2) axis (Figure 3).

Accumulation of AGEs and downstream signaling in skeletal muscle. AGEs bind to RAGE on the muscle cell surface, activating intracellular signaling cascades, including PKC, PI3K/AKT, MAPK/ERK, and JAK/STAT pathways. This leads to NF-κB activation and increased production of reactive oxygen species (ROS) and pro-inflammatory cytokines (TNF-α, IL-1β, IL-6). In parallel, AGEs induce cross-linking of extracellular matrix (ECM) collagen. Together, these processes promote fibrosis and impair muscle function.

### 2.4. Inflammation

In T1DM, insulin deficiency, oxidative stress, AGEs accumulation, and especially chronic hyperglycemia promote the establishment of a systemic inflammatory state that significantly contributes to muscle atrophy. While transient increases in IL-6 can stimulate satellite cell proliferation, chronic and sustained elevations severely impair their function [32,33]. In vitro studies on murine satellite cells have shown that low IL-6 concentrations (0.01–1 ng/mL) dose-dependently enhance proliferation (+31% at 1 ng/mL) via JAK2/STAT3 activation and upregulation of Cyclin D1. However, at higher concentrations (10–100 ng/mL), typically observed in diabetic muscle, this proliferative effect is lost. Persistently high IL-6 levels, as in chronic inflammation associated with diabetes, therefore contribute to satellite cell regenerative failure and exacerbate muscle degeneration [33]. IL-6 activates macrophages and other immune cells and apoptosis-related signaling pathways, further damaging pancreatic β-cells and muscle cells [34]. In skeletal muscle, IL-6 triggers the JAK/STAT3 pathway via binding to IL-6 receptor (IL-6R) and the co-receptor gp130, fostering a chronic inflammatory environment that sensitizes myocytes to apoptosis. Persistent STAT3 activation increases transcription of pro-apoptotic genes such as Fas and Fas Ligand (FasL). The Fas-FasL interaction activates caspase-8, initiating the extrinsic apoptotic cascade, which significantly contributes to muscle mass loss in T1D [35,36]. Concurrently, in pancreatic β-cells, activation of the same pathway facilitates autoimmune destruction and progressive insulin deficiency characteristic of the disease. STAT3 phosphorylation also regulates expression of cytokine signaling suppressors (SOCS), particularly SOCS3, a potent inhibitor of insulin signaling. SOCS3 interferes directly with phosphorylation of IR and insulin receptor substrate 1 (IRS-1) [37,38], thereby blocking PI3K/AKT cascade activation. STAT3 activation also stimulates increased transcription of myostatin, a strong negative regulator of muscle growth. Myostatin, through Smad2/3 signaling, limits satellite cell proliferation and differentiation, further contributing to muscle atrophy [39].

TNF-α, another pro-inflammatory cytokine elevated in T1D patients, directly inhibits the PI3K/AKT pathway [40], thereby blocking mTOR-mediated protein synthesis. It also reduces glucose uptake and utilization in skeletal muscle and adipocytes by decreasing GLUT4 expression [41,42,43]. Mechanistically, TNF-α impairs insulin signaling at multiple levels. De Alvaro et al. [41] demonstrated in neonatal rat myotubes that TNF-α inhibits tyrosine phosphorylation of the insulin receptor and IRS proteins, reducing PI3K activation and AKT phosphorylation, which in turn impairs GLUT4 translocation to the plasma membrane. Consistently, Kaddai et al. [42] reported that TNF-α decreases sortilin, a key regulator of GLUT4 trafficking, further disrupting glucose transport in target tissues. Moreover, phosphorylation of IRS-1 on serine residues (e.g., Ser307) acts as an inhibitory checkpoint, as it prevents subsequent tyrosine phosphorylation required for PI3K/AKT activation. De Alvaro et al. [41] further showed that TNF-α promotes IRS-1 serine phosphorylation via sequential activation of p38 MAPK and IKK-β, thereby disrupting upstream anabolic insulin signaling. Prolonged TNF-α exposure (72–96 h) additionally downregulates IRS-1 and GLUT4 mRNA by >80% and reduces insulin receptor expression by over 50%.

**Figure 4:**
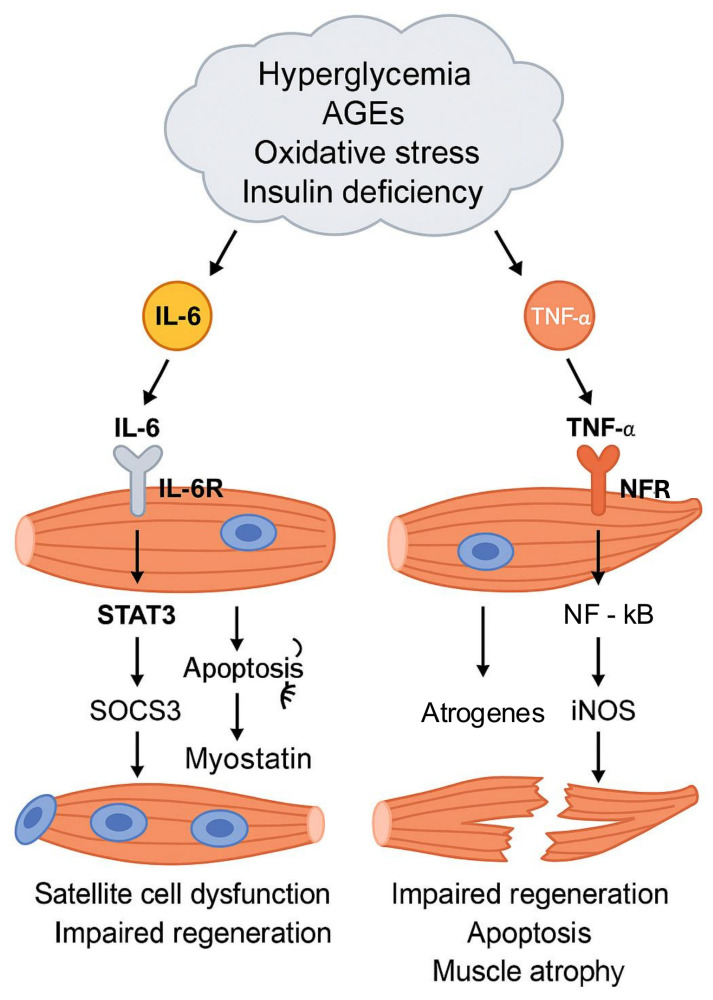
Chronic inflammation drives muscle atrophy in T1D.

Upon binding its receptors on myocyte membranes, TNF-α activates two major downstream signaling pathways: p38 MAPK and IκB kinase (IKK). In muscle, p38 MAPK activates key transcription factors, including NF-κB, which plays a central role in protein metabolism dysregulation [44]. Simultaneously, IKK phosphorylates and promotes degradation of IκB, an inhibitor that normally sequesters NF-κB in the cytoplasm [45]. IκB degradation releases NF-κB, enabling its nuclear translocation and transcriptional regulation of genes involved in muscle atrophy [46]. NF-κB further modulates inducible nitric oxide synthase (iNOS) expression, leading to excessive nitric oxide (NO) production, oxidative stress, and muscle damage [47]. It also upregulates E3 ligases such as MuRF-1 and Atrogin-1, accelerating proteolysis and muscle wasting [44] (Figure 4).

Chronic hyperglycemia, AGEs, oxidative stress, and insulin deficiency promote IL-6 and TNF-α signaling. IL-6 activates JAK/STAT3, inducing SOCS3, myostatin, and Fas/FasL-mediated apoptosis, while TNF-α stimulates p38 MAPK and NF-κB, increasing atrogenes (MuRF-1, Atrogin-1), iNOS, and inhibiting GLUT4 and mTOR signaling. Together, these pathways impair satellite cell function, reduce regeneration, and promote apoptosis, leading to progressive muscle atrophy. (The symbol (≋) behind “apoptosis” represents fragmented DNA).

## 3. Cellular Mechanism of Bone Impairment

In children and adolescents with T1D, bone mass accrual is often compromised, a finding of clinical importance given that peak bone mass, primarily achieved during growth, critically determines skeletal health in adulthood. This impairment reflects the combined effects of endocrine, metabolic, inflammatory, and epigenetic factors, with insulin deficiency, altered anabolic signaling, and chronic hyperglycemia directly impacting bone cells.

### 3.1. Hyperglycemia and Mesenchymal Stromal Cells

**Figure 5:**
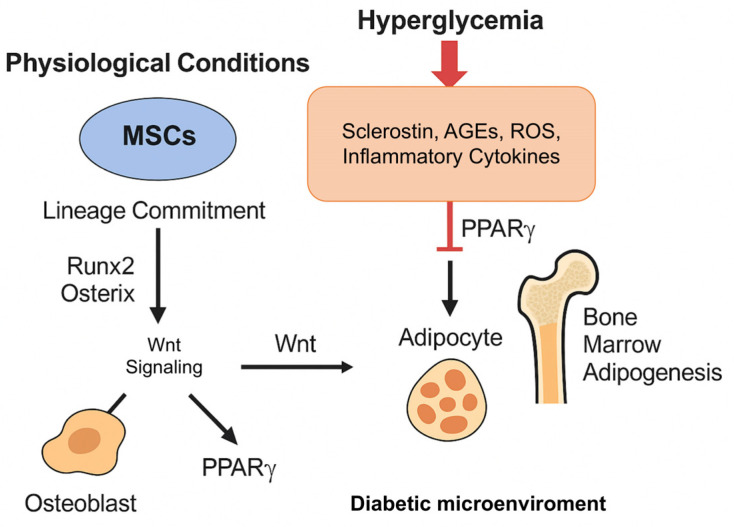
Hyperglycemia-driven imbalance in MSC differentiation.

Mesenchymal stromal cells (MSCs) are the primary progenitors of skeletal tissues, with the ability to differentiate into both osteoblasts and adipocytes. Lineage commitment depends on a balance of transcription factors. Runx2 and Osterix drive osteoblastic differentiation, while peroxisome proliferator-activated receptor gamma (PPARγ) and CCAAT/enhancer-binding protein alpha (C/EBPα) promote adipogenesis [48]. Under physiological conditions, Wnt signaling sustains osteogenesis by suppressing PPARγ and enhancing osteoblastogenic pathways [49]. In the diabetic microenvironment, this balance is profoundly altered. Sclerostin, AGEs, ROS, and inflammatory cytokines inhibit Wnt signaling and reduce Runx2 activity, thereby favoring PPARγ-driven adipogenesis. The consequence is a shift toward bone marrow adipogenesis, with expansion of intramedullary fat at the expense of the osteogenic pool. This remodeling of the marrow niche is not only quantitative. Increased marrow adiposity changes the secretion of adipokines and cytokines, adding pro-inflammatory signals that further suppress osteoblastogenesis and worsen the imbalance in bone remodeling. Functionally, this reduces the capacity to form new bone, impairs physiological turnover, and contributes to skeletal fragility in young patients with T1D [50] (Figure 5).

### 3.2. Insulin and the GH/IGF-1 Axis

**Figure 6:**
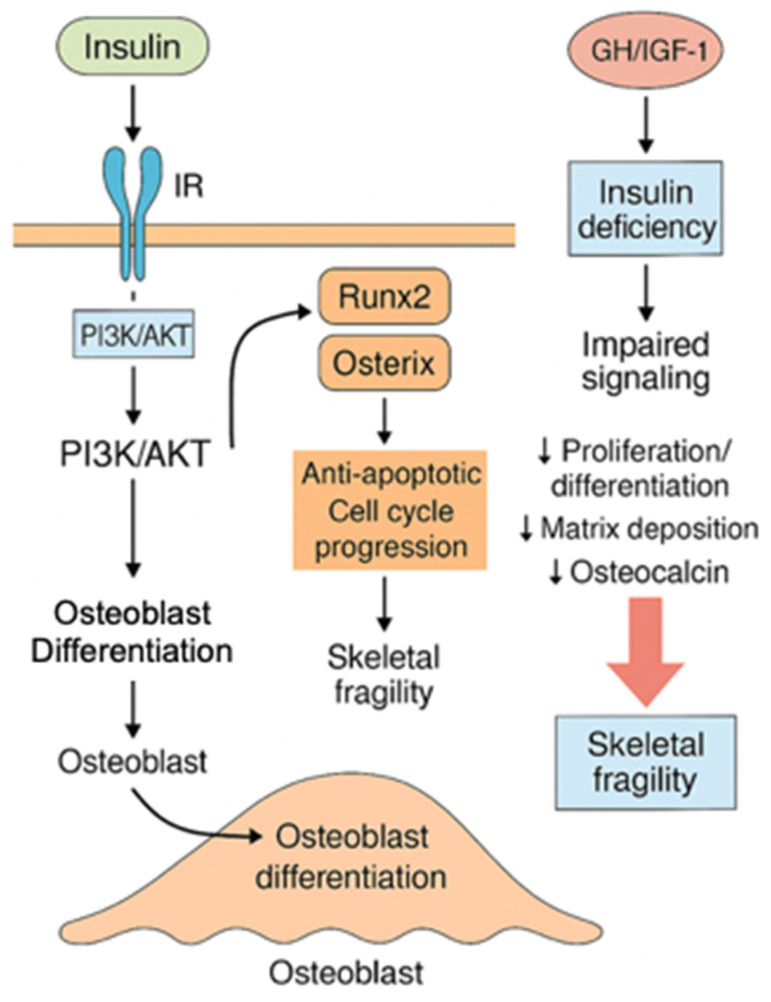
Insulin and GH/IGF-1 signaling in osteoblasts.

In osteoblasts, insulin activates intracellular signaling by engaging its membrane tyrosine kinase receptor. This triggers the PI3K/AKT pathway, which is central to bone anabolism. AKT inactivates GSK3β, stabilizing β-catenin and promoting the transcription of osteogenic genes such as Runx2 and Osterix. AKT also activates Mechanistic Target of Rapamycin Complex 1 (mTORC1), enhancing protein synthesis and type I collagen production, which increases the biosynthetic and functional capacity of osteoblasts. Insulin further supports cell survival by inhibiting pro-apoptotic signals and promoting cell cycle progression. Insulin also stimulates the MAPK/ERK pathway, reinforcing osteoblast differentiation through the upregulation of key transcription factors. In T1D, insulin deficiency impairs these signaling pathways, leading to lower osteoblast proliferation and differentiation, and a decline in matrix deposition, ultimately compromising bone turnover [51]. Reduced osteocalcin production contributes to skeletal dysfunction, as osteocalcin normally enhances insulin secretion and systemic sensitivity, establishing a positive feedback loop that is disrupted in T1D [52]. Growth hormone (GH) and IGF-1 complement insulin in promoting bone growth and anabolism. IGF-1 signals through its tyrosine kinase receptor (IGF1R), activating both PI3K/AKT and MAPK pathways. IGF-1 stimulates collagen synthesis, mineralization, and osteoblast survival. Impaired IGF-1 bioactivity or dysfunction of the GH/IGF-1 axis results in lower proliferation and differentiation of osteogenic populations, further compromising bone formation [51,53] (Figure 6).

Insulin activates PI3K/AKT and MAPK/ERK pathways, promoting Runx2/Osterix activity, collagen synthesis, and osteoblast survival and differentiation. GH/IGF-1 further supports bone anabolism. In T1D, insulin deficiency disrupts these pathways, reducing osteoblast function, osteocalcin production, and bone formation, leading to skeletal fragility. The downward arrow (↓) indicates a decrease or reduction in the corresponding parameter.

### 3.3. Wnt/β-Catenin and RANKL/RANK/OPG Pathways

In diabetes, serum levels of sclerostin and Dickkopf-1 (DKK1), two inhibitors of osteoblastogenesis, are elevated due to metabolic imbalances and inflammatory signals, further dampening Wnt signaling. This reduces Runx2 and Osterix activation, limits collagen and matrix protein synthesis, and constrains the ability of mesenchymal stromal cells to undergo osteogenic differentiation. Together, hyperglycemia, oxidative stress, and inhibitory signals converge into a network that diminishes bone quality, impairing the skeleton’s ability to adapt and regenerate during growth [54].

**Figure 7:**
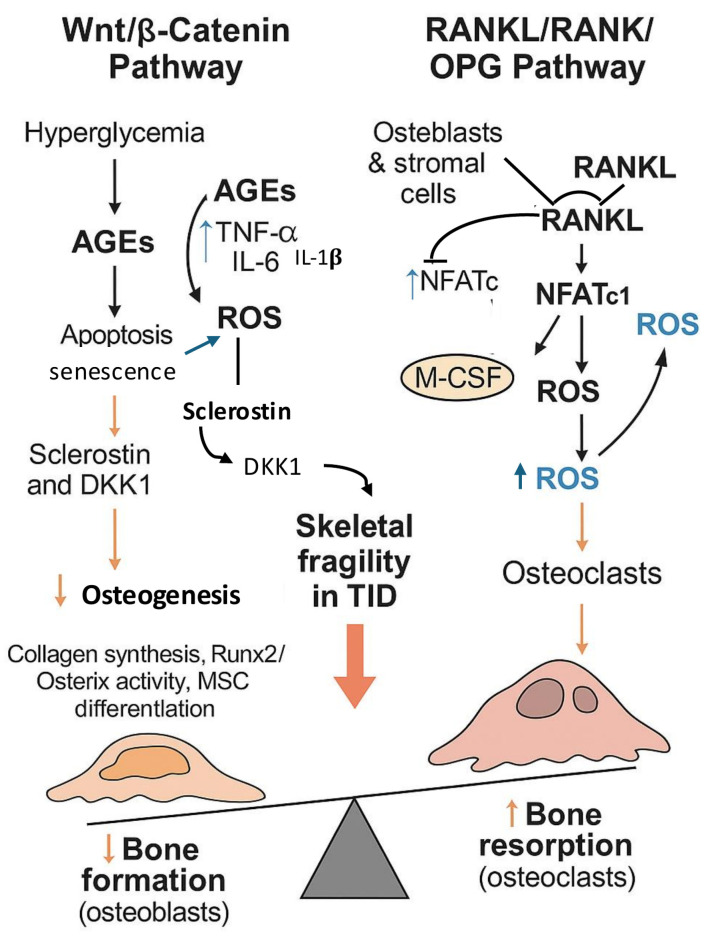
Schematic representation of the molecular mechanisms linking hyperglycemia to impaired bone homeostasis. On the left, the Wnt/β-catenin pathway is suppressed by advanced glycation end-products (AGEs), reactive oxygen species (ROS), and the upregulation of sclerostin and DKK1, leading to reduced osteoblast differentiation, apoptosis, and cellular senescence, ultimately decreasing osteogenesis and bone formation. On the right, the RANKL/RANK/OPG pathway is activated by osteoblasts and stromal cells, promoting osteoclastogenesis via NFATc1 and AP-1 signaling, further enhanced by ROS. The imbalance between diminished bone formation and increased bone resorption contributes to skeletal fragility in type 1 diabetes (T1D). The upward arrow (↑) indicates an increase or activation, while the downward arrow (↓) denotes a decrease or inhibition of the corresponding process or parameter.

In T1D, chronic hyperglycemia, accumulation of AGEs, and oxidative stress disrupt the balance between Receptor Activator of Nuclear factor kappa-B Ligand (RANKL), which promotes osteoclastogenesis, and osteoprotegerin (OPG), a soluble decoy receptor, which counterbalances RANKL activity by binding RANKL and preventing its interaction with RANK. ROS and pro-inflammatory cytokines upregulate RANKL expression and suppress OPG production, amplifying TRAF6, NF-κB, MAPK, and AP-1 signaling, and promoting osteoclast differentiation, fusion, and survival. The cumulative effect is enhanced bone resorption and impaired skeletal integrity, contributing to the skeletal fragility observed in T1D [55] (Figure 7).

### 3.4. “Metabolic Memory” and Mitochondrial Dysfunction

Chronic hyperglycemia and diabetes-associated inflammation led to long-lasting effects on skeletal cells through stable epigenetic modifications. These changes include DNA methylation, histone modifications, and alterations in microRNA (miRNA) expression, which sustain transcriptional programs favoring catabolic or adipogenic pathways even after partial metabolic normalization. This phenomenon establishes a “metabolic memory” that contributes to chronic dysfunction in bone remodeling. Epigenetic enzymes sensitive to the cellular energetic state, such as DNA methyltransferases (DNMT), histone acetyltransferases/deacetylases (HAT/HDAC), and sirtuins, directly link metabolic signals to the transcriptional programs of MSCs and osteoblasts. Concurrently, nutrient excess and oxidative stress compromise mitochondrial function, reducing adenosine triphosphate (ATP) production, increasing ROS generation, and impairing autophagy—the process responsible for the removal of damaged organelles. Insufficient autophagy leads to the accumulation of dysfunctional proteins and organelles, activation of senescence programs, and progressive loss of proliferative and differentiation capacity in bone cells. These processes impair bone regeneration and increase skeletal fragility, establishing a vicious cycle between metabolic alterations and structural deterioration of bone [56].

## 4. Insights from Human Studies on Muscle Skeletal and Bone Impairment

Muscle abnormalities in T1D subjects can occur already after diagnosis, before the development of neuropathic or vascular complications.

A study on muscle biopsy conducted by Dial et al. in T1D physically active subjects and without controls matched for sex, age, and body mass index, demonstrated that, following damaging resistance exercise, individuals with T1D showed impaired strength recovery, increased ECM deposition, a higher frequency of sarcolemma damage, and disruptions in satellite cell content and proliferation [57]. Studies employing handgrip dynamometry in children and adolescents with T1D reported reduced muscle strength and power compared to healthy peers. In a large cross-sectional analysis of 251 children with T1D, Dongare-Bhor et al. demonstrated significantly lower handgrip strength, accompanied by lower total body and lumbar spine bone mineral density (BMD), and reduced trabecular and cortical volumetric BMD assessed by Dual-Energy X-ray absorpiometry (DXA) and peripheral quantitative computed tomography (pQCT) [58]. Importantly, muscle area and trabecular bone density were inversely correlated with HbA1c. A cross-sectional study in a cohort of 95 children and adolescents with T1D evaluated dynamic muscle function using jumping mechanography [59]. Relative muscle power (Pmax/mass) and force (Fmax/body weight) were significantly decreased in T1D suggesting that maximum voluntary muscle power and force related to body weight can be compromised in T1D subjects [59].

Insufficiencies in bone mineral accrual and peak bone mass may contribute to weaker bone structure and higher fracture risk in T1D individuals across the lifespan.

A recent meta-analysis reported a lower bone mineral content (BMC) and areal bone mineral density (aBMD) in T1D subjects compared with controls. Older age and longer disease duration were associated with greater deficits, indicating a cumulative effect of T1D over time [60]. A further meta-analysis evaluating markers of bone formation (osteocalcin, bone alkaline phosphatase) and markers of bone resorption (procollagen I N-propeptide-P1NP and C-terminal Telopeptide of Type I Collagen-CTX) in children and adolescents with T1D showed that osteocalcin was significantly lower in youth with T1D, meaning that osteoblast-driven bone formation appears altered, without clear evidence of increased resorption or altered overall turnover [61].

Chen et al. evaluated bone turnover markers, BMD by DXA, and 3 Tesla-Magnetic Resonance Imaging (MRI) of the proximal tibia to assess bone microarchitecture and vertebral marrow adiposity in T1D subjects compared with age-and sex-matched healthy children [62]. The authors demonstrated that children with T1D had lower levels of bone alkaline phosphatase, and this reduction was more pronounced in those who had poor glucose control or presented with diabetic ketoacidosis (DKA) at diagnosis, suggesting that severe or poorly management of diabetes has a stronger negative impact on bone health. This finding was observed in another study which evaluated sclerostin and DKK-1 in a cohort of T1DM children and adolescents who underwent multiple daily injections (MDI) or continuous subcutaneous insulin infusion (CSII). The authors demonstrated high serum levels of DKK-1 and sclerostin in T1D children, and an improvement of bone health in those on CSII treatment who showed a better glycemic control [63].

Regarding the risk of fractures, a meta-analysis including 39,925 subjects with T1D aged 18–50 years reported a 1.9-fold increased risk compared with non-diabetic individuals [64]. Evidence from a large population-based cohort study using The Health Improvement Network (THIN) in the United Kingdom, which included 30,394 individuals with T1D (aged 0–89 years), demonstrated elevated fracture risk across all age groups [65].

## 5. Prevention and Treatment

Prevention of musculoskeletal impairment in T1D requires a multifaceted approach aimed at optimizing bone and muscle development during the limited window of peak accrual in childhood and adolescence. The primary cornerstone is adequate glycemic control, as higher haemoglobin A1c (HbA1c) levels have been repeatedly linked to lower trabecular BMD, and diminished muscle strength in T1D pediatric cohorts. The target goal is a HbA1c ≤ 6.5% (48 mmol/mol) for children and adolescents with access to advanced diabetes technologies, while in settings without these technologies, the target is ≤7.0% (53 mmol/mol) according to 2024 ISPAD guidelines [66]. Increasing evidence suggests that the greatest long-term complications occur in the presence of pronounced glycemic variability.

Advances in continuous glucose monitoring (CGM) and hybrid closed-loop insulin delivery systems offer the possibility to minimize glycemic variability and chronic hyperglycemia, potentially mitigating the bone accrual deficits observed in vivo.

Moreover, adequate glycemic control is associated with reduced fatigue and a better psychological impact, which are beneficial for engaging in physical activity, another key factor for maintaining proper bone and muscle health [67]. It is well known that moderate-to-vigorous intensity physical activity is strongly recommended in children and adolescents with diabetes (for at least 60 min per day, at least three days per week) for its primary effect of increasing insulin sensitivity, and thereby reducing exogenous insulin requirement. However, levels of physical activity in children and adolescents with T1D are consistently reported to be lower than those observed in their healthy peers, and among the contributing factors is fear of hypoglycemia and stigma among adolescents, which can hinder participation in group activities.

Among the factors to consider for protecting both muscle and bone health, nutrition plays a key role. The International Society for Pediatric and Adolescent Diabetes (ISPAD) recommend a macronutrient distribution consisting of 45–50% carbohydrates, less than 35% fat (with saturated fat <10%), and 15–20% protein [68].

Some studies suggest that patients with T1D often have inadequate calcium intake and reduced 25-OH vitamin D levels. A recent meta-analysis showed that only 27% of T1D subjects achieve sufficient levels [69]. Emerging evidence suggests that vitamin D also plays a pivotal role in skeletal muscle physiology beyond its classical functions in bone metabolism. Reduced serum 25-OH D levels are consistently associated with diminished muscle strength, poorer physical performance, and an increased risk of sarcopenia [70].

Once skeletal or muscular complications have been identified in T1D subject, the therapeutic approach should be tailored. Treatment aims to improve bone mineralization, restore muscle mass and function, and reduce fracture risk, while maintaining optimal glycemic control. Aerobic exercises, such as cycling or swimming, increase cardiovascular fitness and insulin sensitivity, while resistance training promotes muscle hypertrophy and strength, directly addressing the loss of muscle fibers typical of diabetic myopathy. A combination approach has been found to provide the most significant improvements in muscle function and glucose utilization.

These concepts are supported by the findings of Faienza et al. [71], who conducted a cross-sectional study involving 96 children and adolescents with T1D and 34 healthy controls. The study measured bone metabolism markers alongside serum irisin levels, a myokine primarily secreted by skeletal muscle and regulated by physical exercise [72]. The authors demonstrated that T1D patients had significantly higher irisin levels compared to controls, with irisin negatively correlated with HbA1c and disease duration, and positively correlated with markers of bone health.

When the cohort was stratified by insulin therapy, patients treated with CSII displayed the highest irisin concentrations, significantly greater than both those on MDI and healthy peers, suggesting that CSII may enhance glycemic control and bone metabolism through mechanisms involving irisin.

Since T1D adolescents’ physical activity engagement could be challenging, a technological tool like the acT1ve mobile health app could be particularly useful to support safe exercise management, providing real-time, personalized recommendations for insulin and carbohydrate adjustments. As shown in a clinical trial conducted by Shetty et al., such an app not only helps prevent hypoglycemia during and after physical activity (acT1ve is safe and noninferior to standard care regarding hypoglycemia risk) but also increases young people’s awareness of their condition and self-management strategies, especially for those who are newly diagnosed or less experienced with exercise [73].

## 6. Conclusions

T1D disrupts the muscle–bone unit from the earliest phases of life, when peak skeletal and muscular accrual should occur. Converging mechanisms—chronic hyperglycemia, absolute or functional insulin deficiency, AGE–RAGE signaling, low-grade, oxidative stress, and mitochondrial dysfunction—impair satellite cell biology and myogenesis, activate catabolic pathways (FOXO–atrogin-1/MuRF1, UPS), stiffen and remodel the ECM, and blunt bone formation while favoring resorption. Evidence from in vitro, ex vivo, and human studies consistently links these pathways to reduced BMD and microarchitectural quality, diminished muscle mass and performance, and an increase in fracture susceptibility.

Pediatric-onset T1D demands proactive musculoskeletal surveillance integrated with metabolic care. Suboptimal glycemic control, longer disease duration, and microvascular complications are associated with lower BMD, altered trabecular and cortical compartments, decreased bone-formation markers, and poorer muscle strength and power. These changes arise during a narrow developmental window and may set trajectories toward adult osteoporosis and sarcopenia.

Prevention is multifaceted. Tight and stable glycemic control—facilitated by CGM and hybrid closed-loop systems—should be pursued alongside systematic promotion of physical activity (including weight-bearing and progressive resistance training) and nutrition optimized for growth (adequate protein, calcium, and vitamin D, with routine assessment and correction of deficiencies). Exercise is both a metabolic therapy and a mechanobiological stimulus for skeletal muscle; structured, supervised programs can restore strength, improve insulin sensitivity, and enhance functional capacity when deficits are present.

Future priorities include longitudinal pediatric cohorts and randomized trials to define exercise prescriptions across developmental stages, and embed bone–muscle health into routine T1D pathways—from early screening and lifestyle counseling, since an integrated, prevention-first strategy is essential to preserve lifelong musculoskeletal resilience in children and adolescents with T1D.

Regarding possible therapeutic interventions for osteoporosis or fractures, the use of bisphosphonates, drugs used to treat primary and secondary osteoporosis, is not a standard treatment for children with type 1 diabetes due to the lack of long-term safety and efficacy data in this specific population. Although short-term studies have shown potential benefits in animal models by protecting bone cells, longitudinal studies are needed to demonstrate that long-term use of bisphosphonates does not inhibit bone formation.

## Data Availability

No new data were created in this study.

## Abbreviations

The following abbreviations are used in this manuscript:

T1DM	Type 1 Diabetes Mellitus
HbA1c	Hemoglobin A1c
CGM	Continuous Glucose Monitoring
CSII	Continuous Subcutaneous Insulin Infusion
MDI	Multiple Daily Injections
BMD	Bone Mineral Density
BMC	Bone Mineral Content
aBMD	Areal Bone Mineral Density
DXA	Dual-Energy X-ray Absorptiometry
pQCT	Peripheral Quantitative Computed Tomography
AGEs	Advanced Glycation End-products
RAGE	Receptor for Advanced Glycation End-products
ROS	Reactive Oxygen Species
ECM	Extracellular Matrix
PI3K	Phosphoinositide 3-Kinase
AKT	Protein Kinase B
mTOR	Mammalian Target of Rapamycin
FoxO	Forkhead Box O
MuRF1	Muscle-specific RING Finger Protein 1
UPS	Ubiquitin–Proteasome System
IR	Insulin Receptor
IGF-1	Insulin-like Growth Factor 1
IGF1R	Insulin-like Growth Factor 1 Receptor
PGC1α	Peroxisome Proliferator-Activated Receptor Gamma Coactivator 1-Alpha
MAPK	Mitogen-Activated Protein Kinase
ERK	Extracellular Signal-Regulated Kinase
JAK	Janus Kinase
STAT	Signal Transducer and Activator of Transcription
NF-κB	Nuclear Factor Kappa B
TNF-α	Tumor Necrosis Factor Alpha
IL-6	Interleukin-6
IL-1β	Interleukin-1 Beta
SOCS3	Suppressor of Cytokine Signaling 3
GLUT4	Glucose Transporter Type 4
IKK	IκB Kinase
iNOS	Inducible Nitric Oxide Synthase
NO	Nitric Oxide
DKK1	Dickkopf-1
RANKL	Receptor Activator of Nuclear Factor Kappa-B Ligand
RANK	Receptor Activator of Nuclear Factor Kappa-B
OPG	Osteoprotegerin
GH	Growth Hormone
MSCs	Mesenchymal Stromal Cells
PPARγ	Peroxisome Proliferator-Activated Receptor Gamma
C/EBPα	CCAAT/Enhancer-Binding Protein Alpha
AP-1	Activator Protein 1
miRNA	MicroRNA
DNMT	DNA Methyltransferase
HAT	Histone Acetyltransferase
HDAC	Histone Deacetylase
ATP	Adenosine Triphosphate
DKA	Diabetic Ketoacidosis
THIN	The Health Improvement Network
ISPAD	International Society for Pediatric and Adolescent Diabetes
